# Activation of TGF-β Pathway by Areca Nut Constituents: A Possible Cause of Oral Submucous Fibrosis

**DOI:** 10.1371/journal.pone.0051806

**Published:** 2012-12-19

**Authors:** Imran Khan, Neeraj Kumar, Ila Pant, Sivakrishna Narra, Paturu Kondaiah

**Affiliations:** Department of Molecular Reproduction, Development and Genetics, Indian Institute of Science, Bangalore, India; UAE University, United Arab Emirates

## Abstract

Oral submucous fibrosis (OSF) is a chronic inflammatory disease characterized by the accumulation of excess collagen, and areca nut chewing has been proposed as an important etiological factor for disease manifestation. Activation of transforming growth factor-β signaling has been postulated as the main causative event for increased collagen production in OSF. Oral epithelium plays important roles in OSF, and arecoline has been shown to induce TGF-β in epithelial cells. In an attempt to understand the role of areca nut constituents in the manifestation of OSF, we studied the global gene expression profile in epithelial cells (HaCaT) following treatment with areca nut water extract or TGF-β. Interestingly, 64% of the differentially regulated genes by areca nut water extract matches with the TGF-β induced gene expression profile. Out of these, expression of 57% of genes was compromised in the presence of ALK5 (TβRI) inhibitor and 7% were independently induced by areca nut, highlighting the importance of TGF-β in areca nut actions. Areca nut water extract treatment induced p-SMAD2 and TGF-β downstream targets in HaCaT cells but not in human gingival fibroblast cells (hGF), suggesting epithelial cells could be the source of TGF-β in promoting OSF. Water extract of areca nut consists of polyphenols and alkaloids. Both polyphenol and alkaloid fractions of areca nut were able to induce TGF-β signaling and its downstream targets. Also, SMAD-2 was phosphorylated following treatment of HaCaT cells by Catechin, Tannin and alkaloids namely Arecoline, Arecaidine and Guvacine. Moreover, both polyphenols and alkaloids induced TGF-β2 and THBS1 (activator of latent TGF-β) in HaCaT cells suggesting areca nut mediated activation of p-SMAD2 involves up-regulation and activation of TGF-β. These data suggest a major causative role for TGF-β that is induced by areca nut in OSF progression.

## Introduction

Oral submucous fibrosis (OSF) is a chronic inflammatory disease characterized by epithelial atrophy and fibrosis in sub-mucosa of the oral tissues that can cause difficulty in chewing, swallowing, speaking, and mouth opening [1]. Habit of chewing betel nut (*Areca catechu*) has been proposed to be the most important etiological factor in the development of OSF which is also supported by the development of an *in vivo* mouse model with areca nut water extract [2], [3]. Histopathological findings indicate imbalance between synthesis and degradation of extracellular matrix (ECM), mainly of collagen in the oral sub-mucosa leading to OSF [4]. Synthesis of collagen is governed by the balance of pro and anti-fibrogenic cytokines such as transforming growth factor-β (TGF-β), Endothelin-1, Connective tissue growth factor (CTGF) *etc* and Bone morphogenetic protein 4 & 7 (BMP4, 7) respectively [5]. The imbalance leading to over-production of pro-fibrogenic cytokines are known to be associated with fibrosis of different organs [6]. Pro-fibrogenic cytokines become key mediators of fibrosis by differentiating fibroblasts to myofibroblast phenotype in connective tissue disorders [7]. In an earlier report, TGF-β was shown to be up-regulated in OSF tissues [8] and its activation has been shown by the nuclear localization of p-SMAD2 in OSF tissues compared to normals [9], [10]. This activation of TGF-β signaling in OSF tissues could be due to up-regulation of ligand (TGF-β1) and both its activators, αvβ6 integrin and THBS-1. In addition to matrix synthesis, proteases and matrix cross-linking enzymes play important roles in severity of OSF. Alteration in collagen cross-linking makes it resistant to degradation, leading to fibrosis. There are two major collagen cross-linking enzymes proposed in OSF namely; Lysyl oxidase and Transglutaminase 2 [11], [12]. Lysyl oxidase catalyzes formation of aldehydes from lysine residues in collagen and elastin precursors while Transglutaminase-2 (TGM-2) catalyzes transamidating acyltransferase reaction leading to matrix stabilization. These crosslinking enzymes are also known to be affected by the pro-fibrotic cytokines like TGF-β, highlighting the probable role of pro-fibrogenic cytokines in OSF [13], [14]. Taken together, these findings suggest that the TGF-β pathway could possibly play an important role in OSF development. Since betel quid chewing habbit has been proposed to be the most important etiological factor in OSF pathogenesis, several studies were directed towards establishing a role for arecoline, the principal alkaloid present in betel quid, in OSF pathogenesis. Towards this, there have been reports suggesting regulation of TGF-β and its activation by arecoline in epithelial cells [9], [10]. TGF-β activation by arecoline in oral keratinocytes was shown to be through αVβ6 integrin, suggesting an important role for TGF-β in OSF pathogenesis [9]. However, arecoline is approximately 0.2% in areca nut compared to other compounds such as polyphenols, which are approximately 11–17.8% in areca nut [15]. Hence it is possible that in addition to arecoline, other constituents of areca nut extracts may play important roles in OSF pathogenesis. Therefore using a microarray approach, genes differentially regulated by areca nut extract were identified. Interestingly majority of the differentially regulated genes by areca nut water extract were similar to TGF-β regulated genes. Further, the genes regulated by areca extract were also dependent on TGF-β signaling. We also demonstrate that polyphenols and alkaloids in areca nut were able to induce TGF-β signaling by up regulating TGF-β2 and its activator THBS1. Since polyphenols represent a much higher percentage compared to alkaloids in areca nut, these along with other alkaloids could be major etiological factors of OSF pathogenesis involving TGF-β.

## Materials and Methods

### Cell lines and treatments

Primary human gingival fibroblast (hGF) cells were derived from biopsies of Gingival tissues [16] and human keratinocytes (HaCaT) [17] were maintained in DMEM (Sigma-Aldrich, USA) supplemented with 10% fetal bovine serum (Certified grade, Invitrogen corporation, USA. Heat inactivated for HaCaT cells), 100 units/mL penicillin and 100 µg/mL streptomycin (Invitrogen Life Sciences, USA) at 37°C in a humidified chamber with 5% CO2. Human foreskin primary fibroblast cells (FF) (a kind gift by Prof. K. Satyamoorthy, Manipal University, Manipal) were cultured similar to hGF cells as described above. Human Foreskin Keratinocytes (a kind gift by Prof. Annapoorni Rangarajan, IISc) were maintained in Serum-free keratinocyte Medium (Keratinocyte-SFM) supplemented with Bovine Pituitary Extract (BPE 25 µg/ml) and rEGF (Recombinant Epidermal Growth Factor 0.1–0.2 ng/ml). For treatments, cells were serum starved (0.2% serum for hGF and FF cells) for 24 hr and treated with areca nut extracts, different alkaloids (Arecoline 400 µM, Arecaidine 1000 µM, Guvacine 1000 µM), polyphenols (Catechin 170 µM, Tannin 6 µM) (Sigma-Aldrich, USA) or 5 ng/ml TGF-β (R&D systems, USA) for 48 or 72 hrs on HaCaT and hGF cells respectively. For treatment with ALK5 inhibitor (TβRI inhibitor, Sigma-Aldrich, SB 431542), cells were pre-treated with 10 µM of SB 431542 for 2 hr prior to the addition of the respective factors [18].

### RNA extraction, semi-quantitative and real-time RT-PCR

Total RNA was extracted from HaCaT and hGF cells using TRI-reagent (Sigma-Aldrich, St. Louis, USA) according to the manufacturer's protocol. Two micrograms of RNA was reverse transcribed using a cDNA synthesis kit (Applied Biosystems, USA) and 1/100^th^ of the reaction product was used per 20 µL PCR reactions. PCR reactions were performed using DyNAZYME Mastermix (Finnzyme, Finland) in duplicate. PCR products were resolved on a 2% Agarose gel containing Ethidium bromide and the band intensities were determined using a Gel documentation system (UviPro platinum, Uvitec, UK). The expression of RPL-35A gene, a ribosomal protein, whose expression was consistent across treatments in the microarray, was used as a normalizing control [14]. The sequences of primers used in this study are given in table 1.

**Table 1:** List of primer sequences used for RT- PCR.

Sl.No	Genes	F 5′-3′ sequence	R 5′-3′ sequence	Description
1	TGFβ2	AGTGCCTGAACAACGGAT	GTACAAAAGTGCAGCAGG	218 bp, 55°C
2	TGM2	TGACCTCCGCAAAGACAAAG	CCATGACCAGAACAGCAACCT	241 bp, 50°C
3	THBS1	CCGGCGTGAAGTGTACTAGCTA	TGCACTTGGCGTTCTTGTT	317 bp, 59°C
4	TGFBI	TGTGTGCTGAAGCCATCGTTG	CCGGCTTGTCTGAAAAGGTCA	313 bp, 50°C
5	BMP7	AGGCCTGTAAGAAGCACGAG	AGGATGACGTTGGAGCTGTC	268 bp, 59.5°C
6	TMEPAI	TTCATTCCCTGTCCTCATTGG	GCACAACAGCCATGGAATCA	228 bp, 58°C
7	RPL35A	GAACCAAAGGGAGCACACAG	CAATGGCCTTAGCAGGAAGA	236 bp, 58°C
8	COL1A1	TCCCCAGCCACAAAGAGTCTA	TTTCCACACGTCTCGGTCA	201 bp, 58°C
9	COL2A1	ACCCTGAGTGGAAGAGTGGA	CCACCATTGATGGTTTCTCC	199 bp, 58°C
10	COL1A3	TTGACCCTAACCAAGGATGC	GGAAGTTCAGGATTGCCGTA	201 bp, 58°C
11	α-SMA	CAGCCAAGCACTGTCAGG	CAATGGATGGGAAAACAGC	150 bp, 59.5°C

Real time PCR quantitations were performed in ABI Prism 7000 sequence detection system and analysed with SDS 2.1 software (Applied Biosystems, USA). The reactions were identical as described above except that Dynamo™ SYBERgreen 2× mix (Finnzymes, Finland) was used in place of DyNAZYME mix in triplicate reactions. RPL-35A expression was used for normalization and the differential expression was determined by the formula
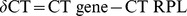


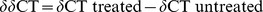


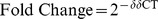



### Microarray protocols and data analysis

Microarray experiments were performed using Whole human genome (4×44 k) oligonucleotide arrays (Agilent Technologies, Santa Clara, USA). For labeling reactions, 200 ng of RNA each from untreated and treated samples were used. Labeling of the probes was done using the Low RNA Input Linear Amplification Kit (Agilent Technologies, USA) according to the manufacturer's protocol. The image analysis was done using Feature extraction tool version 9.5.3.1 (Agilent Technologies) and data analysis was done using Bioconductor LIMMA package. The background-corrected raw intensity values were used for analysis. LOWESS algorithm was used to normalize the data and fold change (Fc) was calculated based on the ratio of Cy5/Cy3 (treated/untreated) intensities. For statistical analysis, Lmfit and eBayes (Empirical Bayes method) test was performed and P value correction was performed using Benjamini Hochberg method [19]. First commonly regulated genes between areca nut water extract (5H) and TGF-β was found out (using a P value≤0.05 and fold change cut off of ≥1.5). To find out genes that were compromised upon areca nut treatment (5H) in presence of ALK5 inhibitor, ALK5 inhibitor array intensity was taken as control and the differentially regulated genes were identified in the 5H+ALK5 inhibitor arrays. All the microarray data have been submitted to GEO database and accession number is GSE38227.

### Preparation of Areca nut extracts and fractionation

Areca nut extract preparation and fractionation were performed according to previously described methods [20], [21]. Briefly thirty grams of dried and de-husked Betel nut was ground and extracted by 100 ml of de-ionized water for 4 h at 4°C with constant stirring. After filtration with a sintered glass funnel, these extracts were lyophilized and re-dissolved in de-ionized water. Insoluble components were further extracted with ethanol by the same procedure as above. All the extracts were then re-filtered through a 0.2 µm filter, divided into aliquots, lyophilized and stored at 4°C. For treatments, the weighed dry powder was dissolved in de-ionized water and stored at −70°C.

Filtered water extract samples were partitioned with dichloromethane in the ratio of 1∶1 by volume. Then the water phase was collected and the impurities associated with dichloromethane were also collected (**DCM phase**). The partitioning with dichloromethane was repeated for 3 times and the water phase was further partitioned with ethyl acetate (1∶1 by volume), which was also repeated for 3 times. The ethyl acetate extracts were collected and evaporated to dryness with vacuum rotary evaporator (Buchi Rotavapor model R- 210, Switzerland) before analysis. All the three phases namely Dichloromethane (DCM phase), water (Alkaloid phase) and ethyl acetate (Polyphenol phase) were used for treating cells. To check the cross contamination between alkaloid and polyphenol extracts, LC-MS profiling of areca nut alkaloid and polyphenol fractions were performed (Figure S1). The above LC-MS profile's retardation time and m/z values were matched with the known predominant alkaloid (Arecoline) and polyphenol (catechin) of areca nut. Arecoline peak was found to be present only in alkaloid fraction and Catechin peak was found to be present only in polyphenol fractions, highlighting the purity of fractions with respect to major alkaloids and polyphenols (Figure S2).

### Western blot analysis

Proteins were extracted from cells after DPBS wash using lysis buffer (Tris-Hcl 50 mM, NaCl 150 mM, SDS 0.1%, NP-40 0.5%, and protease inhibitor cocktail, Sigma Aldrich, USA). Equal amount of protein (determined by Bradford method) extracted from cells were resolved on 12% SDS-PAGE gel, transferred to polyvinylidene difluoride membrane and subjected to immunoblot analysis. To block nonspecific binding sites, blots were incubated in 5% non-fat dry milk for 1 h followed by overnight incubation in primary antibodies at 4°C diluted according to the manufacturer's instructions (p-SMAD2, Cell Signaling Technology, Massachusetts, 3101, dilution 1∶1000; Total SMAD2, Cell Signaling Technology, Massachusetts, 3162, dilution 1∶1000; TGM2, R&D SYSTEMS, MAB3542, dilution 1∶500; β-Actin, Sigma-Aldrich, C2206, dilution 1∶2000). This was followed by incubation with horseradish peroxidase-conjugated secondary antibody (Anti-rabbit/mouse HRP conjugated secondary antibody Sigma-Aldrich, St. Louis, MO, USA, dilution 1∶2000). Proteins were visualized with a chemiluminescence detection system (Super Signal West Femto Chemiluminescent Substrate, Thermo Fisher Scientific Inc. USA, 34095) and subsequent exposure to X-ray film.

### TGF-β2 Immunoassay

TGF-β2 in the spent medium of areca nut treated cells were measured using commercially available Quantikine human TGF-β2 immunoassay kit (R&D Systems, Minneapolis, MN, USA). Briefly, HaCaT cells spent medium untreated/treated with different concentrations of areca nut were activated by using Hydrochloric acid (1 N) for 10 min and later mixed with HEPES (0.5 M)/NaOH (1.2 N) buffer followed by dilution in calibrator diluent. Standards and samples are pipetted into the wells pre-coated with TGF-β2 monoclonal antibody and allowed to bind for 2 hrs at ambient temperature. After washing away unbound substances, an enzyme-linked polyclonal antibody specific for TGF-β2 was added to the wells and washed with buffer to remove any unbound antibody-enzyme reagent. Following this, substrate solution was added to the wells and colour development was allowed for 20 min, stopped by adding stop buffer and the intensity of the colour was measured at 450 nm after wavelength correction at 570 nm. Concentration of TGF-β2 was calculated from the standard curve.

### Total collagen staining using Direct Red 80

Direct Red 80 was purchased from Sigma-Aldrich, USA and the staining protocol followed has been described earlier [22]. Direct Red 80 dye was dissolved in saturated aqueous picric acid at a concentration of 100 mg/100 ml. Bouin's fluid (for cell fixation) was prepared by mixing 15 ml saturated aqueous picric acid with 5 ml 35% formaldehyde and 1 ml glacial acetic acid. After treatment of hGF cells, cells were washed with PBS, fixed with 1 ml Bouin's fluid for 1 hr followed by washing with PBS for 15 min. Cells were air dried before adding 1 ml Direct Red dye reagent for 1 h under mild shaking on a microplate shaker. Later on dye solution was removed followed by washing in 0.01 N hydrochloric acid to remove excess dye. The stained cells were photo documented before dissolving the stain. For quantitation, 1 ml of 0.1 N NaOH was added to 35 mm culture dishes followed by incubation for 30 min at room temperature on microplate shaker. The dye solution's optical density (OD) was measured by spectrophotometer (Bio-Rad, SmartSpec-3000 Spectrophotometer) at 550 nm against 0.1 N NaOH as a blank and OD/10^5^ cells were plotted.

### Statistical analysis

Statistical significance was determined using one-way analysis of variance (ANOVA) among different treatments. All treatments were compared to untreated and the observed significance levels from multiple comparisons were made using the Bonferroni's Multiple Comparison Test with *P*≤0.05 indicating significance (P value≤0.01, ≤0.001 and ≤0.0001 are indicated by _*_, _**_, and _***_ respectively).

## Results

### Areca nut induces TGF-β signaling in epithelial cells

**Figure 1:**
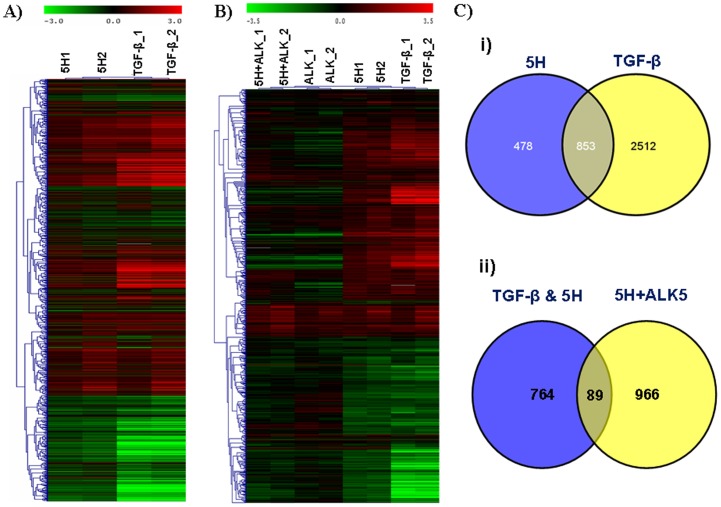
Hierarchical clustering of TGF-β and Areca nut induced genes. A] Hierarchical clustering of commonly regulated genes in HaCaT cells following treatments with areca nut water extract (5H−5 µg/ml) or TGF-β (5 ng/ml) (P≤0.05 and ≥1.5 fold). B] Hierarchical cluster of genes that are commonly regulated by areca nut or TGF-β but compromised in presence of ALK5 inhibitor. Red, green and black colours in A and B represent up, down or un- regulated genes respectively. The vertical axis represents genes and horizontal axis represents treatments. C] i and ii, Venn diagram representation of the genes from A and B respectively.

**Figure 2:**
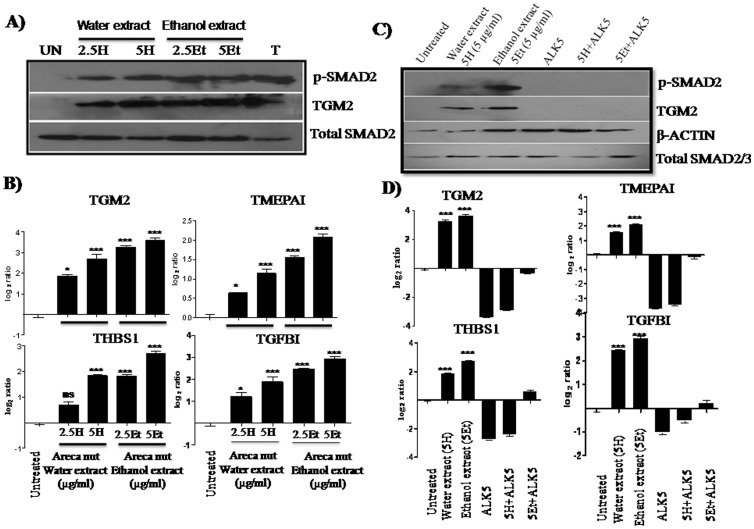
Areca nut induces TGF-β signaling in epithelial cells. A & B] Areca nut induced genes by Western blot and by qRT-PCR. HaCaT cells were serum starved for 24 hours and treated with either areca nut water (H) or ethanol (Et) extracts 2.5, 5 µg/ml (2.5H/5H & 2.5Et/5Et) in serum free medium for 48 hours. A) Western blot of HaCaT cell lysates showing induction of p-SMAD2 and its downstream target TGM2. B) qRT-PCR analysis showing expression of TGF-β down-stream target genes TGM2, TMEPAI, THBS1 and TGFBI. **UN**-Untreated, 2.5H & 5H− 2.5 and 5 µg/ml Areca nut water extract, 2.5 Et,& 5Et- 2.5 and 5 µg/ml, **T**-TGF-β (_***_ = P<0.0001 compared to untreated). C & D] ALK5 inhibitor (SB 431542) reverses the Areca nut induced expression of genes in HaCaT cells. HaCaT cells were treated with areca nut water (H) and ethanol (Et) extracts (5H− 5 µg/ml areca nut water extract and 5Et- 5 µg/ml areca nut ethanol extract) in the presence or absence of ALK5 (TβRI) inhibitor. Western blot of p-SMAD2 and TGM2 (C). qRT-PCR of TGM2,TMEPAI, THBS1 and TGFBI (D). Untreated, 5H− Areca nut water extract (5 µg/ml), 5Et- Areca nut Ethanol extract (5 µg/ml), **ALK5**- TβRI inhibitor, 5H+ALK5 inhibitor, 5Et+ALK5 inhibitor (_***_ = P≤0.0001 compared to untreated).

In order to study the areca nut effects on epithelial cells, gene expression profiling was performed using Agilent whole human genome 4×44 K arrays with the RNAs extracted from HaCaT cells treated with areca nut water extract (5 µg/ml; 5H). 1331 genes were found to be differentially expressed following treatment with areca nut water extract (P≤0.05, fold change ≥1.5). KEGG pathway analysis revealed regulation of genes categorized in several pathways and among them prominent were pathways in cancer, cell cycle, cytokine-cytokine receptor interaction, Wnt signaling pathway etc (Table 2). Interestingly many of these genes are targets of TGF-β signaling [23], which include TGF-β2, SMAD3, FN1, MMP1, MMP2, MMP9, CCNE1 *etc*. In a previous microarray study on differentially expressed genes in OSF, we and others proposed activation of TGF-β pathway as a major cause of OSF pathogenesis. In view of this, and regulation of TGF-β pathway genes by areca nut extract, we hypothesized that areca nut actions could be through the activation of TGF-β signaling. To test this, we compared expression profile of differentially regulated genes in HaCaT cells by areca nut water extract and TGF-β. Treatment of HaCaT cells by areca nut extract and TGF-β resulted in the regulation of 1331 and 3365 genes respectively. Of areca nut induced genes, 853 genes (64%) matched with TGF-β regulated genes (Figure 1A & Ci, Table S1). List of top 30 genes up and down regulated by areca nut extract along with commonly regulated genes by areca nut extract and TGF-β are shown in table 3 and 4, respectively. A high match in differentially regulated genes by areca nut extract and TGF-β suggests a possible activation of TGF-β signaling by areca nut extract. To test this, HaCaT cells were treated with areca nut water extract in the presence of ALK5 inhibitor (TβRI inhibitor, SB431542) and gene expression profiling was compared with areca nut extract regulated genes. Out of the 853 commonly expressed genes between areca nut water extract and TGF-β, 764 genes were compromised by SB431542 treatment (Figure 1Cii), highlighting the role of TGF-β in areca nut actions. Hierarchical cluster analysis of the differentially regulated genes following the above treatments, confirmed common gene expression profiles by areca nut and TGF-β (Figure 1B). The above results demonstrate areca nut inducing/activating TGF-β signaling in epithelial cells. To confirm this we treated HaCaT cells with areca nut water extract in the presence or absence of ALK5 inhibitor and activation of TGF-β pathway was studied. By western blot analysis, we observed phosphorylation of SMAD2 (an effector of TGF-β signaling) after treatment with areca nut water extract (Figure 2A). Also, protein expression of TGM2 (a TGF-β induced gene) was found to be induced by areca nut extracts, suggesting activation of TGF-β pathway by areca nut extract. Further, by qPCR, expression of TGF-β target genes (TGM2, TMEPAI, THBS1 and TGFBI) was found to be up regulated following treatment with areca nut extracts (Figure 2B). All these effects of areca nut were compromised in the presence of ALK5 inhibitor (Figure 2C and D). Similar results were obtained using ethanol extract of water insoluble fraction of areca nut suggesting similar action of both water and ethanol extracts of areca nut on epithelial cells. Taken together, regulation of majority of the genes by areca nut in epithelial cells involves TGF-β signaling. To confirm these results in another cell line, human foreskin keratinocytes were treated with areca nut extracts (described in the methods) and the expression profile of TGF-β pathway genes was studied. Like HaCaT cells, areca nut treatment on foreskin keratinocytes was able to induce all the TGF-β target genes which also get compromised in presence of TβRI inhibitor, suggesting areca nut actions through TGF-β (Figure S3A–D).

**Table 2:** Pathways differentially expressed upon areca nut treatment on HaCaT cells as revealed by KEGG pathway analysis.

Pathways	No of Genes	Pathways	No of Genes
hsa01100 Metabolic pathways	55	hsa04142 Lysosome	13
hsa05200 Pathways in cancer	34	hsa04010 MAPK signaling pathway	13
hsa05166 HTLV-I infection	25	hsa04062 Chemokine signaling pathway	13
hsa04110 Cell cycle	24	hsa05152 Tuberculosis	13
hsa05034 Alcoholism	22	hsa05169 Epstein-Barr virus infection	13
hsa05322 Systemic lupus erythematosus	21	hsa04115 p53 signaling pathway	13
hsa05202 Transcriptional misregulation in cancer	19	hsa05145 Toxoplasmosis	12
hsa04060 Cytokine-cytokine receptor interaction	18	hsa04141 Protein processing in endoplasmic reticulum	12
hsa04114 Oocyte meiosis	18	hsa04722 Neurotrophin signaling pathway	12
hsa04510 Focal adhesion	18	hsa04726 Serotonergic synapse	11
hsa04144 Endocytosis	16	hsa04120 Ubiquitin mediated proteolysis	11
hsa05164 Influenza A	16	hsa04145 Phagosome	11
hsa04810 Regulation of actin cytoskeleton	14	hsa05146 Amoebiasis	11
hsa04310 Wnt signaling pathway	13	hsa04912 GnRH signaling pathway	10

**Table 3:** List of top 30 genes showing Up or Down regulation upon areca nut treatment on HaCaT cells.

Up Regulated Genes			Down Regulated Genes		
Gene Name	Genbank accession	Fold Change	Gene Name	Genbank accession	Fold Change
VIM	NM_003380	5.28	CCL2	NM_002982	3.99
KRT16	NM_005557	4.12	PIF1	NM_025049	3.37
AK021467	AK021467	4.1	A_32_P49116	A_32_P49116	3.34
SERPINB3	NM_006919	4.01	BIRC3	NM_001165	3.19
SERPINB4	NM_002974	3.99	LCN2	NM_005564	3.16
C8orf68	BC022082	3.88	IFI44L	NM_006820	2.99
AF334588	AF334588	3.82	CLEC2B	NM_005127	2.99
AKR1C1	NM_001353	3.8	A_23_P369966	A_23_P369966	2.79
A_24_P610387	A_24_P610387	3.68	CA9	NM_001216	2.76
MFAP5	NM_003480	3.62	IFIT3	NM_001549	2.75
AKR1C3	NM_003739	3.61	FAM72D	NM_207418	2.72
SRPX	NM_006307	3.54	ELF3	NM_004433	2.65
A_24_P934592	A_24_P934592	3.54	BE138567	BE138567	2.65
FCRLA	NM_032738	3.45	ASPM	NM_018136	2.61
TRIM49	NM_020358	3.42	KIF23	NM_138555	2.59
SCN2B	NM_004588	3.22	APOBEC3B	NM_004900	2.59
GK	NM_203391	3.14	UBE2C	NM_181803	2.58
HMOX1	NM_002133	3.07	LOC283711	XR_040656	2.58
SERPINE2	NM_006216	3.03	A_23_P76480	A_23_P76480	2.54
A_32_P69987	A_32_P69987	3.03	BUB1B	NM_001211	2.52
A_23_P135634	A_23_P135634	2.98	CDKN3	NM_005192	2.51
DOCK4	NM_014705	2.96	TPX2	NM_012112	2.5
A_24_P934989	A_24_P934989	2.89	CENPA	NM_001809	2.47
LOC151438	AK055877	2.88	NMU	NM_006681	2.47
ACAA2	NM_006111	2.85	TROAP	NM_005480	2.47
MAF	NM_001031804	2.83	BIRC5	NM_001012271	2.44
ANO7	NM_001001891	2.83	CENPE	NM_001813	2.41
HSPB3	NM_006308	2.79	A_32_P188921	A_32_P188921	2.41
BAAT	NM_001701	2.78	NUF2	NM_145697	2.41
VNN3	NR_028290	2.77	SPAG5	NM_006461	2.4

**Table 4:** List of top 30 commonly regulated genes showing Up or Down regulation by TGF-β and areca nut treatment on HaCaT cells.

Up Regulated Genes				Down Regulated Genes			
Gene Name	Genbank accession	TGF-β	5H	Gene Name	Systematic Name	TGF-β	5H
PDPN	NM_006474	15.91	2.41	UBE2C	NM_181803	17.87	2.58
MAF	NM_001031804	13.44	2.83	BIRC5	NM_001012271	16.70	2.30
ACAA2	NM_006111	13.40	2.85	CEP55	NM_018131	15.50	2.00
MMP13	NM_002427	13.27	1.52	ASPM	NM_018136	15.28	2.61
A_23_P123234	A_23_P123234	10.49	2.21	DEPDC1	NM_017779	14.72	2.64
SERPINE1	NM_000602	9.68	1.45	NDC80	NM_006101	13.71	1.91
CST6	NM_001323	9.54	2.37	PBK	NM_018492	13.64	1.91
FCRLA	NM_032738	8.67	3.45	BUB1	NM_004336	13.35	1.83
PDZK1	NM_002614	8.25	1.81	NCAPG	NM_022346	13.29	1.74
VIM	NM_003380	8.16	5.28	SPAG5	NM_006461	12.71	2.40
A_32_P115518	A_32_P115518	8.04	1.50	CDCA5	NM_080668	12.70	1.84
PRSS23	NM_007173	7.98	1.74	CDT1	NM_030928	12.64	1.65
VASN	NM_138440	7.78	1.78	TTK	NM_003318	12.57	2.09
BAMBI	NM_012342	7.67	1.78	MND1	NM_032117	12.49	1.73
A_23_P87421	A_23_P87421	7.50	1.51	NUF2	NM_145697	11.98	2.41
KRT16	NM_005557	7.39	4.12	BUB1B	NM_001211	11.74	2.52
TGM2	NM_004613	7.31	1.81	KIF23	NM_138555	11.44	2.59
HILS1	NR_024193	7.26	1.50	FAM72D	NM_207418	11.41	2.72
TPM1	NM_000366	7.25	1.62	SKA3	BC013418	11.35	1.74
WNT5B	NM_030775	7.08	1.53	HMGB2	NM_002129	11.27	2.37
MFAP5	NM_003480	6.68	3.62	KIF18B	BC044933	10.67	2.35
SERPINE2	NM_006216	6.42	3.03	FAM83D	NM_030919	10.29	2.00
ALOX5	NM_000698	6.37	2.13	TOP2A	NM_001067	10.06	2.04
A_32_P52414	A_32_P52414	6.15	2.19	CCNA2	NM_001237	9.90	2.14
LOC151438	AK055877	6.12	2.88	KIF2C	NM_006845	9.88	2.02
A_24_P633825	A_24_P633825	6.11	2.18	CENPA	NM_001809	9.83	2.47
HSPB3	NM_006308	5.83	2.79	CIT	NM_007174	9.73	1.89
DACT1	NM_016651	5.56	1.51	CDC2	NM_001786	9.69	1.89
SLITRK6	NM_032229	5.53	1.67	FAM64A	NM_019013	9.64	1.86
SPOCK1	NM_004598	5.47	2.42	LMNB1	NM_005573	9.42	1.95

### Areca nut alkaloids and polyphenols induce TGF-β signaling

**Figure 3:**
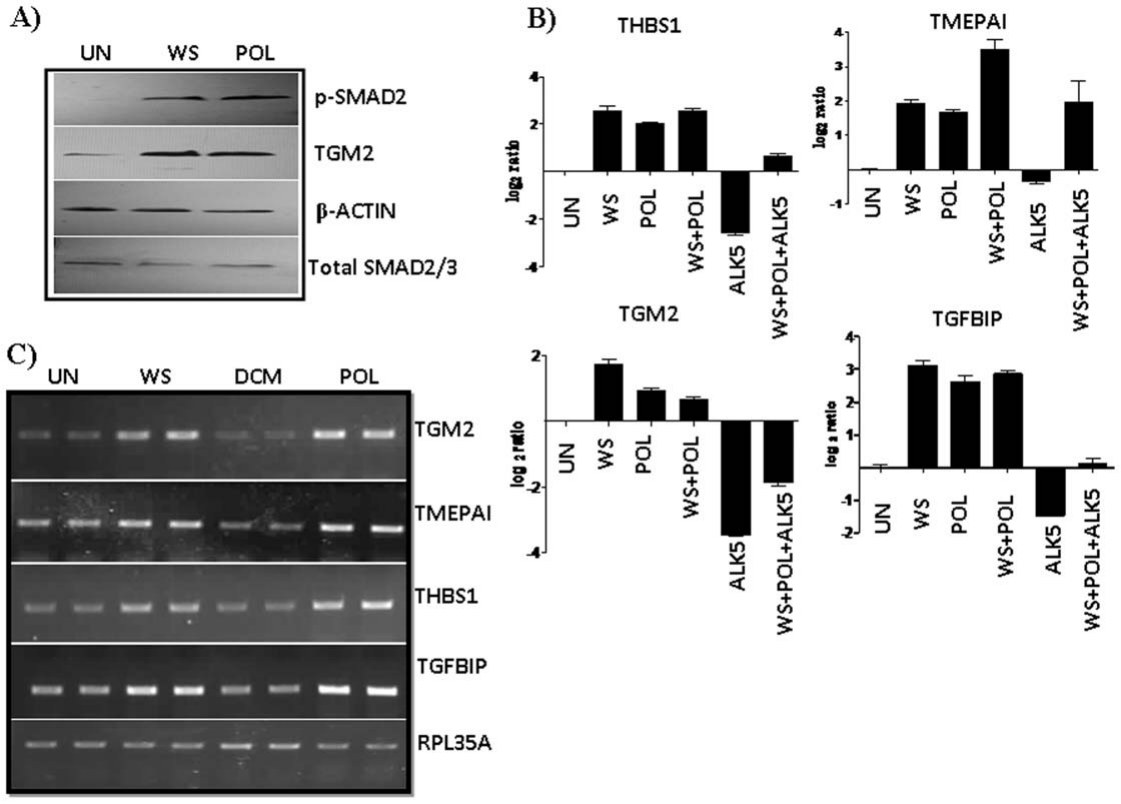
Alkaloid and Polyphenol fractions of Areca nut induce TGF-β signaling in HaCaT cells. Treatment of HaCaT cells with both the Alkaloid and Polyphenol fractions of areca nut water extract induced TGF-β signaling (p-SMAD2) and its down-stream target TGM2 as shown by the western blot (Figure 3A). Expression of TGF-β down-stream targets were also studied by Real Time PCR (Figure 3B) and semi quantitative PCR (Figure 3C). Induction of genes by alkaloid and polyphenol fractions of areca nut was compromised in presence of TβRI inhibitor (ALK5 inhibitor). (UN- untreated, WS- water supernatant, POL- Polyphenol supernatant, DCM- Dichloromethane fraction).

**Figure 4:**
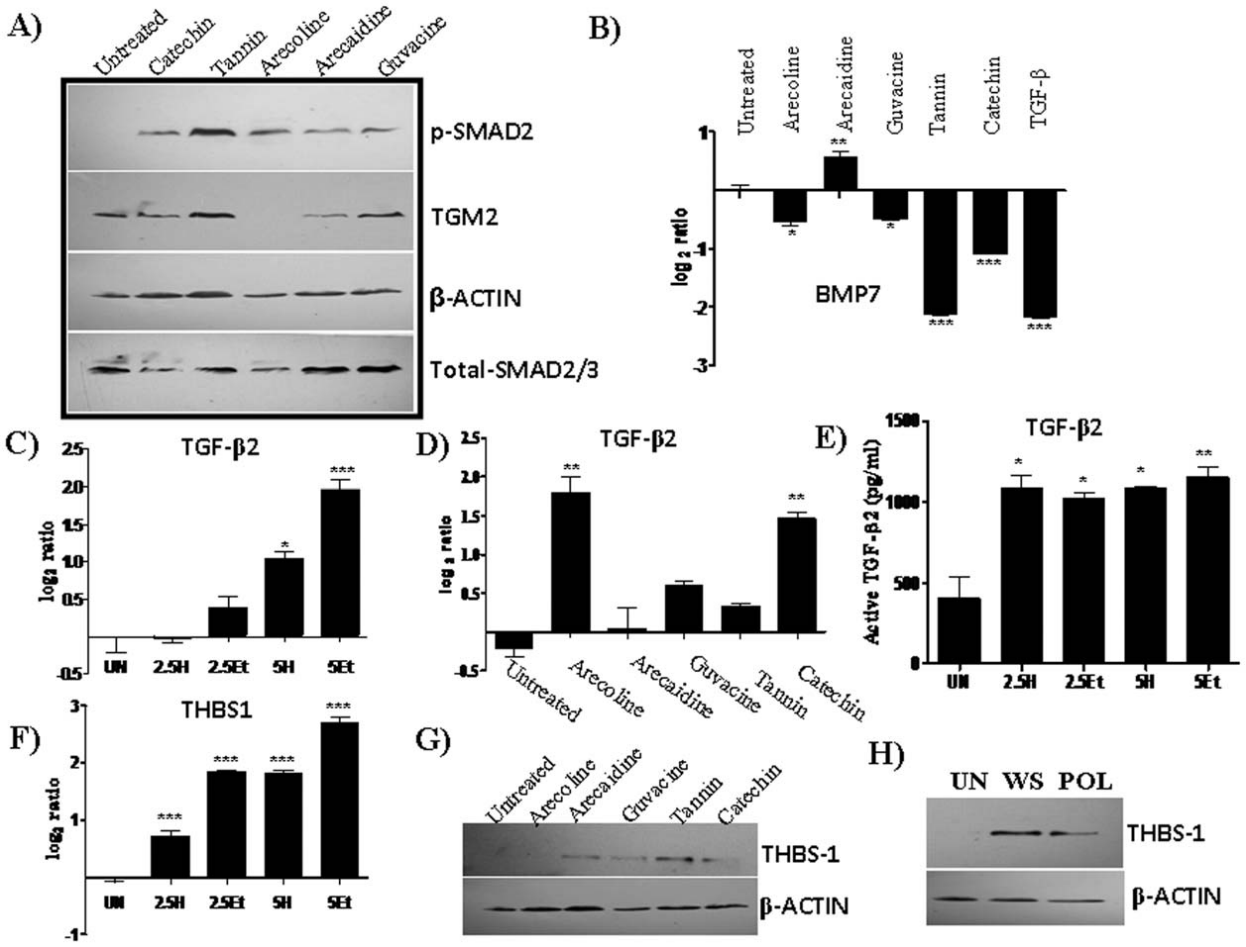
Both the pure alkaloids and polyphenols of areca nut induce TGF-β signaling. Human keratinocytes (HaCaT) cells were serum deprived for 24 hours and treated with areca nut extracts, pure Alkaloids and Polyphenols for 48 hrs. A, shows pSMAD2 by western blot and B, expression of BMP7 by real time PCR; C & D, TGF-β2 expression by real time PCR; E, TGF-β2 protein estimation by ELISA; F, THBS1 expression by real time PCR; G & H, THBS1 western blots. The treatments are depicted in the respective figures. UN- untreated, Arecoline (400 µM), Arecaidine (1000 µM), Guvacine (1000 µM), Catechin (170 µM), Tannin (6 µM), TGF-β,, WS- water supernatant, POL- Polyphenol supernatant.

In order to find out areca nut components responsible for inducing TGF-β signaling, fractionation of areca nut water extract was performed as described in materials and methods. Upon treatment of areca nut fractions (alkaloids and polyphenols) on HaCaT cells, both the alkaloid and polyphenol fractions induced phosphorylation of SMAD2, expression of TGF-β down-stream targets – TGM2, THBS1, TGFBI and TMEPAI which were compromised in the presence of ALK5 inhibitor (Figure 3A–C). Similar results were observed in foreskin keratinocytes cells where treatment of both the areca nut fractions ( alkaloids and polyphenol) were able to induce TGF-β down-stream targets which gets compromised in presence of TβRI inhibitor (Figure S3E–H). Constituents of areca nut alkaloid and polyphenol fractions are well defined [21]. Arecoline is the most predominant alkaloid (∼0.2%) followed by Arecaidine, Guvacine etc and Catechin and Tannin are the most predominant polyphenols present along with minor polyphenols like Epicatechin, Gallic acid etc [21]. In order to determine which alkaloid or polyphenol of areca nut induce TGF-β signaling, we treated HaCaT cells with pure alkaloids (Arecoline, Arecaidine and Guvacine) and pure polyphenols (Catechin and Tannin). Similar to water and polyphenol fractions of areca nut extract; arecoline, arecaidine, guvacine, catechin and tannin also induced phosphorylation of SMAD2, expression of down-stream target TGM2 (Figure 4A). In contrast, the anti-fibrogenic cytokine BMP7 [10] was found to be down-regulated following the above treatments (Figure 4B). In addition, treatment of HaCaT cells with water and ethanol extracts, and pure compounds resulted in the induction of TGF-β2 RNA and protein (Fig. 4 C–E). Also, THBS1 RNA and protein were induced by above treatments (Figure 4F–H). Therefore induction of TGF-β signaling by areca nut and its constituent in epithelial cells is by inducing both ligand (TGF-β2) and its activator, THBS1.

### Areca nut does not induce TGF-β signaling in fibroblast cells

During chewing of betel quid, epithelium is first exposed to areca nut components and later it gets diffused into sub-mucosal region. Therefore, areca nut components may affect both epithelial cells as well as connective tissue consisting of fibroblasts that may be important for the pathogenesis of OSF. Hence we also studied the actions of areca nut extracts on human gingival fibroblasts. In contrast to its action on epithelial cells, areca nut water extract did not induce p-SMAD2 and its down-stream targets in human gingival fibroblast (hGF) cells (Figure S4A & B). Similarly, treatment of hGF cells with alkaloid, DCM and polyphenol fractions of areca nut water extract did not show any induction of TGF-β target genes (Figure S4C). These observations suggest that areca nut influence on fibroblasts may not be as profound as compared to epithelial cells in the induction of pro fibrogenic cytokines.

### Areca nut potentiates TGF-β action on hGF cells

**Figure 5:**
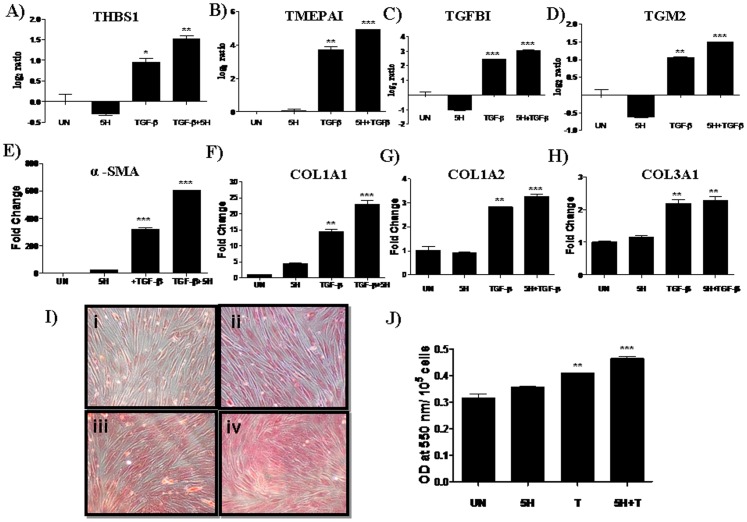
Areca nut potentiates TGF-β action on hGF cells. Human gingival fibroblast (hGF) cells were serum deprived by replenishing with medium containing 0.2% serum for 24 h. Subsequently, hGF cells were treated for 72 hours with areca nut water extract (5H− 5 µg/ml) and or TGF-β (5 ng/ml). Real time PCR was used to examine the expression of genes regulated by TGF-β or areca nut extracts (A, B, C, D, E, F, G and H). I] Direct Red 80 stained hGF cells showing the presence of total collagens following treatment with Areca nut water extract and/or TGF-β for 5 days (Figure 5I, 10× magnification images, i-Untreated, ii-5H (5 µg/ml) Areca nut water extract, iii- TGF-β, iv- 5H+TGF-β). Figure 5J shows the quantitation of the Direct Red staining as measured at 550 nm.

In response to areca nut components, TGF-β induced by the epithelium can diffuse into connective tissues and effect fibrotic changes in the sub-mucosa. Since areca nut did not activate TGF-β pathway in fibroblasts, we studied the combined actions of areca nut and TGF-β on fibroblasts. Interestingly, upon exposure of fibroblast cells (both hGF and FF) to areca nut extract in the presence of TGF-β, there was enhanced expression of TGF-β target genes, THBS-1, TMEPAI, TGFBI and TGM-2 (Figure 5 A–D & Figure S5 A–D). This suggests potentiation of TGF-β action by areca nut water extract on fibroblasts. Furthermore, in response to areca nut and TGF-β, the expression of α-SMA and collagen isoforms (Col1A1, 1A2 and Col3A1) was found to be synergistic and additive, respectively (Figure 5 E–H). Similar results were also observed in FF cells upon treatments of areca nut and TGF-β (Figure S5 E–H). This additive effect of TGF-β and areca nut on collagen expression was also observed for total collagen protein as revealed by Direct Red dye staining in both hGF and FF cells (Figure 5 I, J & Figure S5 I).

## Discussion

Etiology of OSF is highly complicated and involves intrinsic and extrinsic factors including habit of betel quid chewing coupled with predisposition of the subjects for developing OSF [24]. Several etiological factors have been proposed but arecoline present in the betel nut has been shown to be the most important alkaloid implicated in OSF development [25]. In previous reports including ours, up-regulation and activation of TGF-β signaling in OSF has been demonstrated [9], [10]. This activation of TGF-β involves up-regulation of αVβ6 integrins by arecoline. The above findings highlight the role of arecoline in TGF-β activation and a central role for TGF-β pathway in the development of OSF. But the amount of arecoline needed to induce TGF-β signaling and its down-stream targets was much higher (50 µg/ml or 400 µM) than the amount of areca nut water extract (2.5 µg/ml). This led us to speculate that areca nut constituents other than arecoline could also be involved in inducing TGF-β signaling in epithelial cells.

We demonstrate for the first time that both water and alcohol extracts of areca nut induce TGF-β signaling in epithelial cells as shown by increased levels of p-SMAD2. This could be due to induction of TGF-β ligand (TGF-β2) and its activator THBS-1 leading to activation of TGF-β pathway. Accordingly, areca nut induced expression shares a large number of genes (64%) regulated by TGF-β. In the TGF-β signaling cascade, TGF-β ligands (1–3) bind to TβRII receptor that recruits TβRI (ALK5) leading to phosphorylation of ALK5 that facilitates SMAD2/3 phosphorylation. Activated SMAD2 or SMAD3 demonstrates activation of TGF-β signaling. Inhibition of ALK5 leads to abrogation of SMAD 2/3 phosphorylation [18].. Therefore we used an ALK5 inhibitor to demonstrate TGF-β's role in areca nut actions. As expected, SMAD2 phosphorylation following areca nut treatment was completely abolished in the presence of ALK5 inhibitor and consequently majority of areca nut regulated genes (57%) were also compromised. This suggests that areca nut regulated gene expression in HaCaT cells involves TGF-β signaling. It is also evident that there are genes that are regulated by areca nut independent of TGF-β signaling (∼36%). As of now, the role of these genes in the OSF pathogenesis is not known.

We also establish that in addition to arecoline, other alkaloids such as arecaidine and guvacine are able to induce TGF-β signaling. Most importantly, both the predominant polyphenols of areca nut (Catechin and Tannin) were found to be potent inducers of TGF-β signaling. This is a very profound finding that the alkaloids, other than arecoline and predominant polyphenols of areca nut are potent inducers of pro fibrogenic cytokine, TGF-β. Polyphenols constitute 11–17.8% in mature areca nut compared to its predominant alkaloid arecoline (0.2%) [21]. This higher percentage of polyphenols in areca nut could play a dominant role in inducing pro-fibrogenic (TGF-β) pathway and consequently, OSF.

**Figure 6:**
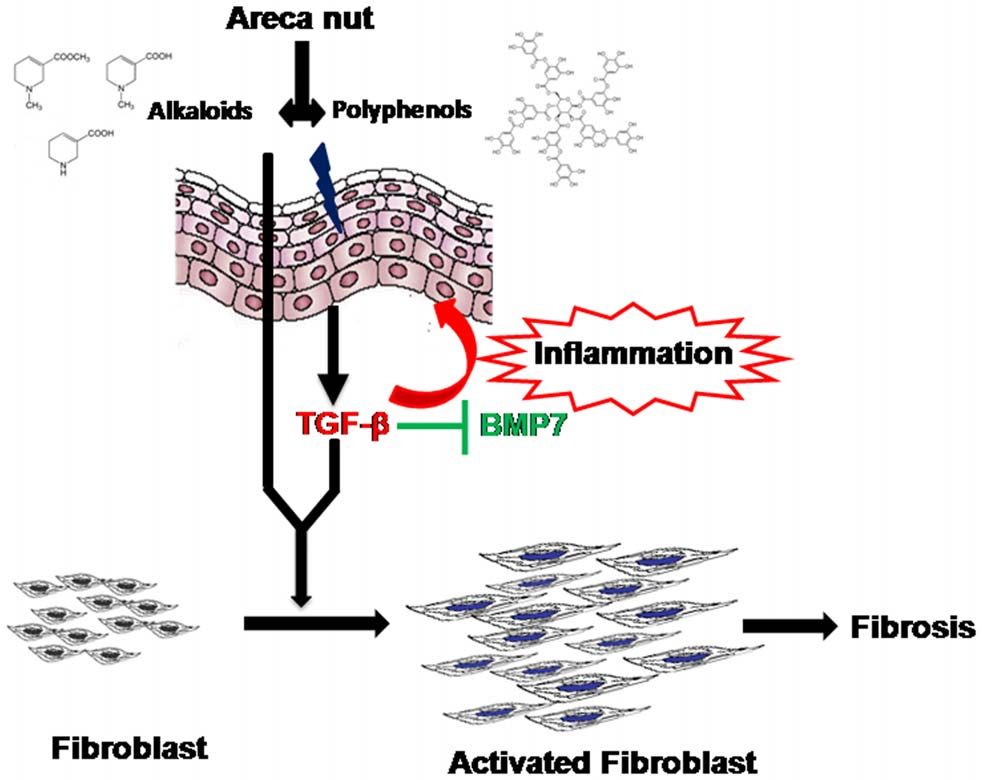
Diagrammatic representation of proposed model of OSF pathogenesis by areca nut and its constituents. Areca nut first comes in contact with epithelial cells where it's both the constituent, alkaloids and polyphenols acts on the epithelial cells and induces TGF-β signaling. This induced TGF-β signaling in the epithelial cells could be source of inflammation and can also diffuse into the connective tissue where it suppresses anti-fibrogenic cytokines like BMP7. In the connective tissue, areca nut acts on fibroblast cells along with TGF-β produced from the epithelium and potentiates its action in activating fibroblast cells responsible for inducing fibrosis.

Our studies revealed that areca nut has very little direct influence on the expression of pro fibrogenic genes in the fibroblasts, where the actual disease process occurs. However, our data advocates that TGF-β induced in the epithelium by areca nut acts on the fibroblasts in a pro fibrogenic manner by the induction of matrix components such as collagens. This is also supported by our previous results showing hGF cells if cultured in HaCaT spent medium treated with arecoline, induced collagen isoforms which gets neutralized by LAP (Latency Associated peptide, a TGF-β antagonist) [26]. Most interestingly, although areca nut has minimal influence on fibroblasts, it synergizes with TGF-β in activating fibroblast cells. This is very important in the context of OSF manifestation in susceptible individuals. Chronic areca nut chewing may trigger pro-inflammatory/fibrotic factors from epithelium and these factors may synergize with areca nut leading to fibroblast activation, where the disease process occurs (Figure 6).

In conclusion, in this manuscript we propose a causative role for areca nut components in triggering a pro fibrogenic cascade involving TGF-β pathway from the epithelial cells influencing the underlying sub mucosa for a fibrotic response. For the first time we show the potent induction of TGF-β pathway by polyphenols and alkaloids in addition to arecoline present in areca nut extract.

## Supporting Information

Figure S1 **Liquid chromatography of alkaloid and polyphenol fractions.** Areca nut water extract was partitioned into two phases namely, Ethyl acetate (Polyphenol) fraction and Water supernatant (Alkaloid) fraction. To asses the purity of fractions both the above fractions were separated in HPLC. Figure S1 A&B shows the retardation profile of the two fractions which does not match with each other highlighting both have different components.
(TIF)

Figure S2 **Identification of major components of alkaloid and polyphenol fractions by LC–MS.** In order to see the distribution of areca nut major alkaloids and polyphenols in the Ethyl acetate and water supernatant fractions, MS Spectrum of pure Arecoline and pure catechin were matched with the above two fractions. Arecoline MS profile (m/z- 79, 156.76) matched with water supernatant (Figure S2 A) but not with ethyl acetate fraction. Similarly Catechin MS profile (m/z 279, 579 and 867) matched with ethyl acetate fraction (Figure S2 B) but not with water supernatant, highlighting no cross contamination of major alkaloid and polyphenol in the fractionation.
(TIF)

Figure S3 **Areca nut does not induce TGF-β signaling in fibroblast cells.** Human gingival fibroblast (hGF) cells were treated for 72 hours with areca nut water (H) or ethanol (Et) extract 2.5, 5 µg/ml (2.5H/5 H & 2.5 Et/5 Et etc) in 0.2% serum containing medium for 72 hours. Figure S3A shows the western blot of the above treatments on hGF cells where only TGF-β (5 ng/ml) induces p-SMAD2. A semi-quantitative PCR was also performed with the different concentration of areca nut water, ethanol extract and with the purified fractions of the areca nut water extract treated on hGF cells but fail to induce TGF-β down-stream targets (Figure S3 B& C). (Untreated, 2.5H− 2.5 µg/ml, 5H− 5 µg/ml of areca nut water extract, 2.5 Et & 5Et- 2.5 and 5 µg/ml of areca nut ethanol extract, UN- Untreated, Ws- Water supernatant, POL- Polyphenol).
(TIF)

Figure S4 **Arecanut induces TGF-β signaling in foreskin keratinocytes through TGF-β.** Human Foreskin keratinocytes cells were serum starved for 24 hours and treated with either areca nut water (H) or ethanol (Et) extracts 2.5, 5 µg/ml (2.5H/5H & 2.5Et/5Et) in serum free medium for 48 hours. A-D) qRT-PCR analysis showing expression of TGF-β down-stream target genes TGM2, TMEPAI, THBS1, TGFBI and ALK5 (TβRI) inhibitor (SB 431542) reverses the Areca nut induced expression of the above genes. Treatment of human Foreskin keratinocytes cells with both the Alkaloid and Polyphenol fractions of areca nut water extract induced TGF-β down-stream target as shown by Real Time PCR (Figure S4E-H) and induction of genes by alkaloid and polyphenol fractions of areca nut was compromised in presence of TβRI inhibitor. **UN**-Untreated, 2.5H & 5H− 2.5 and 5 µg/ml Areca nut water extract, 2.5 Et,& 5Et- 2.5 and 5 µg/ml, ALK5- ALK5 inhibitor, **T**-TGF-β, WS- water supernatant, POL- Polyphenol supernatant, DCM- Dichloromethane fraction. (_***_ = P<0.0001, compared to untreated).
(TIF)

Figure S5 **Areca nut potentiates TGF-β action on FF cells.** Human foreskin fibroblast (FF) cells were treated for 72 hours with areca nut water extract (5H− 5 µg/ml) and or TGF-β (5 ng/ml) after 24 hrs of serum deprivation in 0.2% serum and the expression of genes were looked at by Real-Time PCR (Figure S5 A–H). FF cells were treated with areca nut water extract and or TGF-β for 3 days and stained with “Direct Red 80” for total collagen. Figure S5 I shows the quantitation of the Direct Red staining as measured at 550 nm.
(TIF)

Table S1 List of commonly regulated genes by Areca nut water extract (5H) and TGF-β (p≤0.05; Fold Change 1.5).
(XLS)

## References

[1] Le PV, Gornitsky M, Domanowski G (1996) Oral stent as treatment adjunct for oral submucous fibrosis. Oral Surg Oral Med Oral Pathol Oral Radiol Endod 81: 148–150. 8665305 10.1016/s1079-2104(96)80404-5

[2] Sinor PN, Gupta PC, Murti PR, Bhonsle RB, Daftary DK, et al (1990) A case-control study of oral submucous fibrosis with special reference to the etiologic role of areca nut. J Oral Pathol Med 19: 94–98. 2341977 10.1111/j.1600-0714.1990.tb00804.x

[3] Sumeth Perera MW, Gunasinghe D, Perera PA, Ranasinghe A, Amaratunga P, et al (2007) Development of an in vivo mouse model to study oral submucous fibrosis. J Oral Pathol Med 36: 273–280. 17448137 10.1111/j.1600-0714.2007.00523.x

[4] Rajalalitha P, Vali S (2005) Molecular pathogenesis of oral submucous fibrosis–a collagen metabolic disorder. J Oral Pathol Med 34: 321–328. 15946178 10.1111/j.1600-0714.2005.00325.x

[5] Zeisberg M, Hanai J, Sugimoto H, Mammoto T, Charytan D, et al (2003) BMP-7 counteracts TGF-beta1-induced epithelial-to-mesenchymal transition and reverses chronic renal injury. Nat Med 9: 964–968. 12808448 10.1038/nm888

[6] Ihn H (2002) Pathogenesis of fibrosis: role of TGF-beta and CTGF. Curr Opin Rheumatol 14: 681–685. 12410091 10.1097/00002281-200211000-00009

[7] Krieg T, Abraham D, Lafyatis R (2007) Fibrosis in connective tissue disease: the role of the myofibroblast and fibroblast-epithelial cell interactions. Arthritis Res Ther 9 Suppl 2: S4. 10.1186/ar2188 PMC2072888 17767742

[8] Haque MF, Meghji S, Khitab U, Harris M (2000) Oral submucous fibrosis patients have altered levels of cytokine production. J Oral Pathol Med 29: 123–128. 10738939 10.1034/j.1600-0714.2000.290304.x

[9] Moutasim KA, Jenei V, Sapienza K, Marsh D, Weinreb PH, et al (2011) Betel-derived alkaloid up-regulates keratinocyte alphavbeta6 integrin expression and promotes oral submucous fibrosis. J Pathol 223: 366–377. 21171082 10.1002/path.2786

[10] Khan I, Agarwal P, Thangjam GS, Radhesh R, Rao SG, et al (2011) Role of TGF-beta and BMP7 in the pathogenesis of oral submucous fibrosis. Growth Factors 29: 119–127. 21591998 10.3109/08977194.2011.582839

[11] Trivedy C, Warnakulasuriya KA, Hazarey VK, Tavassoli M, Sommer P, et al (1999) The upregulation of lysyl oxidase in oral submucous fibrosis and squamous cell carcinoma. J Oral Pathol Med 28: 246–251. 10426196 10.1111/j.1600-0714.1999.tb02033.x

[12] Thangjam GS, Agarwal P, Khan I, Verma UP, Balapure AK, et al (2009) Transglutaminase-2 regulation by arecoline in gingival fibroblasts. J Dent Res 88: 170–175. 19278990 10.1177/0022034508329633

[13] Shanley CJ, Gharaee-Kermani M, Sarkar R, Welling TH, Kriegel A, et al (1997) Transforming growth factor-beta 1 increases lysyl oxidase enzyme activity and mRNA in rat aortic smooth muscle cells. J Vasc Surg 25: 446–452. 9081125 10.1016/s0741-5214(97)70254-4

[14] Ranganathan P, Agrawal A, Bhushan R, Chavalmane AK, Kalathur RK, et al (2007) Expression profiling of genes regulated by TGF-beta: differential regulation in normal and tumour cells. BMC Genomics 8: 98. 17425807 10.1186/1471-2164-8-98 PMC1858692

[15] IARC (2004) Betel-quid and areca-nut chewing and some areca-nut derived nitrosamines. IARC Monogr Eval Carcinog Risks Hum 85: 1–334. 15635762 PMC4781453

[16] Nigam M, Ranjan V, Srivastava S, Sharma R, Balapure AK (2008) Centchroman induces G0/G1 arrest and caspase-dependent apoptosis involving mitochondrial membrane depolarization in MCF-7 and MDA MB-231 human breast cancer cells. Life Sci 82: 577–590. 18279897 10.1016/j.lfs.2007.11.028

[17] Boukamp P, Petrussevska RT, Breitkreutz D, Hornung J, Markham A, et al (1988) Normal keratinization in a spontaneously immortalized aneuploid human keratinocyte cell line. J Cell Biol 106: 761–771. 2450098 10.1083/jcb.106.3.761 PMC2115116

[18] Inman GJ, Nicolas FJ, Callahan JF, Harling JD, Gaster LM, et al (2002) SB-431542 is a potent and specific inhibitor of transforming growth factor-beta superfamily type I activin receptor-like kinase (ALK) receptors ALK4, ALK5, and ALK7. Mol Pharmacol 62: 65–74. 12065756 10.1124/mol.62.1.65

[19] Smyth GK (2004) Linear models and empirical bayes methods for assessing differential expression in microarray experiments. Stat Appl Genet Mol Biol 3: Article3. 16646809 10.2202/1544-6115.1027

[20] Jeng JH, Kuo ML, Hahn LJ, Kuo MY (1994) Genotoxic and non-genotoxic effects of betel quid ingredients on oral mucosal fibroblasts in vitro. J Dent Res 73: 1043–1049. 8006230 10.1177/00220345940730050501

[21] IARC Working Group on the Evaluation of Carcinogenic Risks to Humans (2004) Betel-quid and areca-nut chewing and some areca-nut derived nitrosamines. IARC Monogr Eval Carcinog Risks Hum 85: 1–334. 15635762 PMC4781453

[22] Tullberg-Reinert H, Jundt G (1999) In situ measurement of collagen synthesis by human bone cells with a sirius red-based colorimetric microassay: effects of transforming growth factor beta2 and ascorbic acid 2-phosphate. Histochem Cell Biol 112: 271–276. 10550611 10.1007/s004180050447

[23] Zavadil J, Bitzer M, Liang D, Yang YC, Massimi A, et al (2001) Genetic programs of epithelial cell plasticity directed by transforming growth factor-beta. Proc Natl Acad Sci U S A 98: 6686–6691. 11390996 10.1073/pnas.111614398 PMC34413

[24] Tilakaratne WM, Klinikowski MF, Saku T, Peters TJ, Warnakulasuriya S (2006) Oral submucous fibrosis: review on aetiology and pathogenesis. Oral Oncol 42: 561–568. 16311067 10.1016/j.oraloncology.2005.08.005

[25] Harvey W, Scutt A, Meghji S, Canniff JP (1986) Stimulation of human buccal mucosa fibroblasts in vitro by betel-nut alkaloids. Arch Oral Biol 31: 45–49. 3458437 10.1016/0003-9969(86)90112-3

[26] Thangjam GS, Agarwal P, Balapure AK, Rao SG, Kondaiah P (2009) Regulation of extracellular matrix genes by arecoline in primary gingival fibroblasts requires epithelial factors. J Periodontal Res 44: 736–743. 19438976 10.1111/j.1600-0765.2008.01185.x

