# Supplementary figures and images for: Activation of TGF-β Pathway by Areca Nut Constituents: A Possible Cause of Oral Submucous Fibrosis
Source: PLoS One. 2012 Dec 19;7(12):e51806. doi: 10.1371/journal.pone.0051806 (PMC3526649; doi:10.1371/journal.pone.0051806)

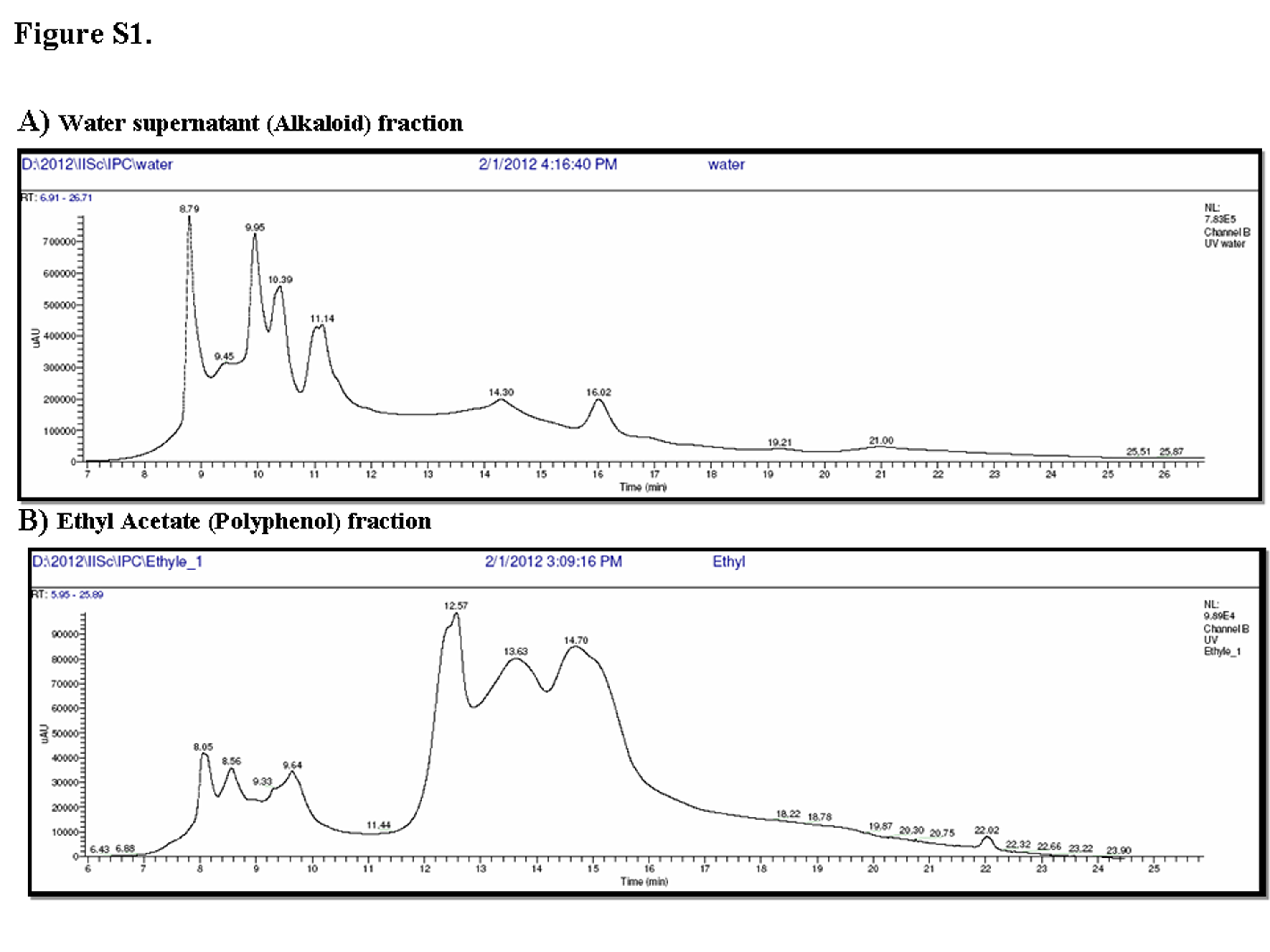

Supplement: Figure S1 — Liquid chromatography of alkaloid and polyphenol fractions. Areca nut water extract was partitioned into two phases namely, Ethyl acetate (Polyphenol) fraction and Water supernatant (Alkaloid) fraction. To asses the purity of fractions both the above fractions were separated in HPLC. Figure S1 A&B shows the retardation profile of the two fractions which does not match with each other highlighting both have different components. (TIF) [file pone.0051806.s001.tif]

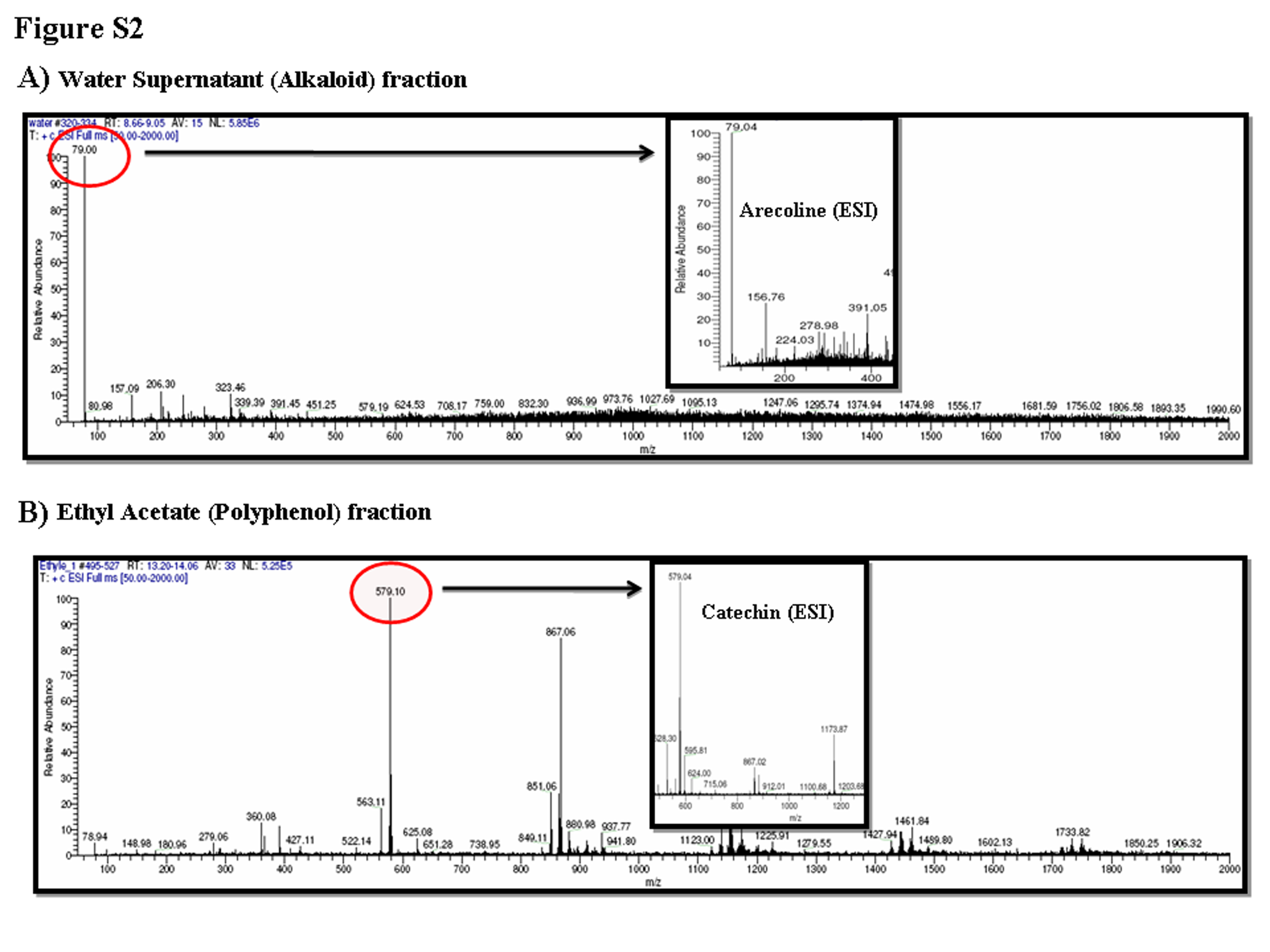

Supplement: Figure S2 — Identification of major components of alkaloid and polyphenol fractions by LC–MS. In order to see the distribution of areca nut major alkaloids and polyphenols in the Ethyl acetate and water supernatant fractions, MS Spectrum of pure Arecoline and pure catechin were matched with the above two fractions. Arecoline MS profile (m/z- 79, 156.76) matched with water supernatant (Figure S2 A) but not with ethyl acetate fraction. Similarly Catechin MS profile (m/z 279, 579 and 867) matched with ethyl acetate fraction (Figure S2 B) but not with water supernatant, highlighting no cross contamination of major alkaloid and polyphenol in the fractionation. (TIF) [file pone.0051806.s002.tif]

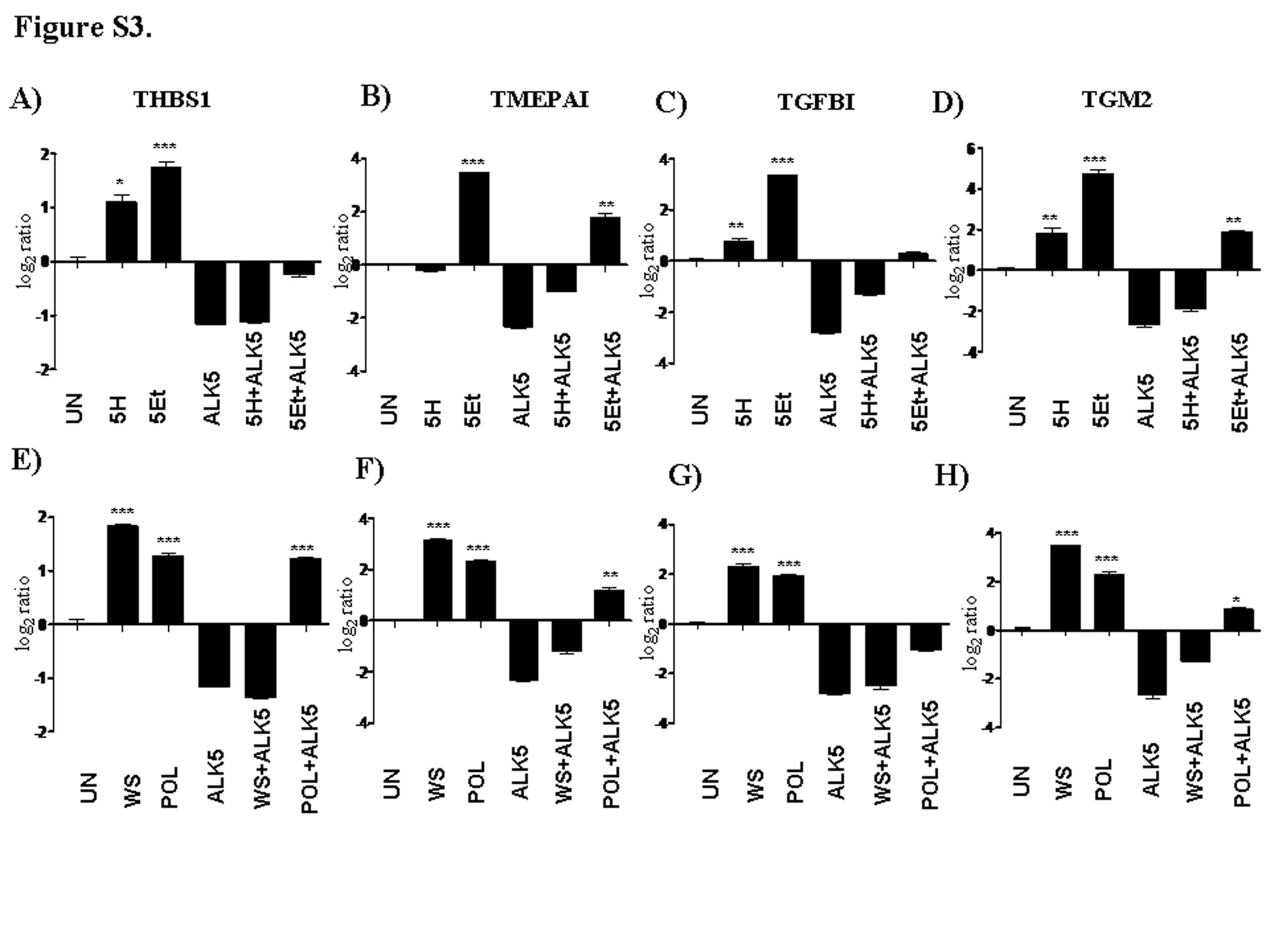

Supplement: Figure S3 — Areca nut does not induce TGF-β signaling in fibroblast cells. Human gingival fibroblast (hGF) cells were treated for 72 hours with areca nut water (H) or ethanol (Et) extract 2.5, 5 µg/ml (2.5H/5 H & 2.5 Et/5 Et etc) in 0.2% serum containing medium for 72 hours. Figure S3A shows the western blot of the above treatments on hGF cells where only TGF-β (5 ng/ml) induces p-SMAD2. A semi-quantitative PCR was also performed with the different concentration of areca nut water, ethanol extract and with the purified fractions of the areca nut water extract treated on hGF cells but fail to induce TGF-β down-stream targets (Figure S3 B& C). (Untreated, 2.5H− 2.5 µg/ml, 5H− 5 µg/ml of areca nut water extract, 2.5 Et & 5Et- 2.5 and 5 µg/ml of areca nut ethanol extract, UN- Untreated, Ws- Water supernatant, POL- Polyphenol). (TIF) [file pone.0051806.s003.tif]

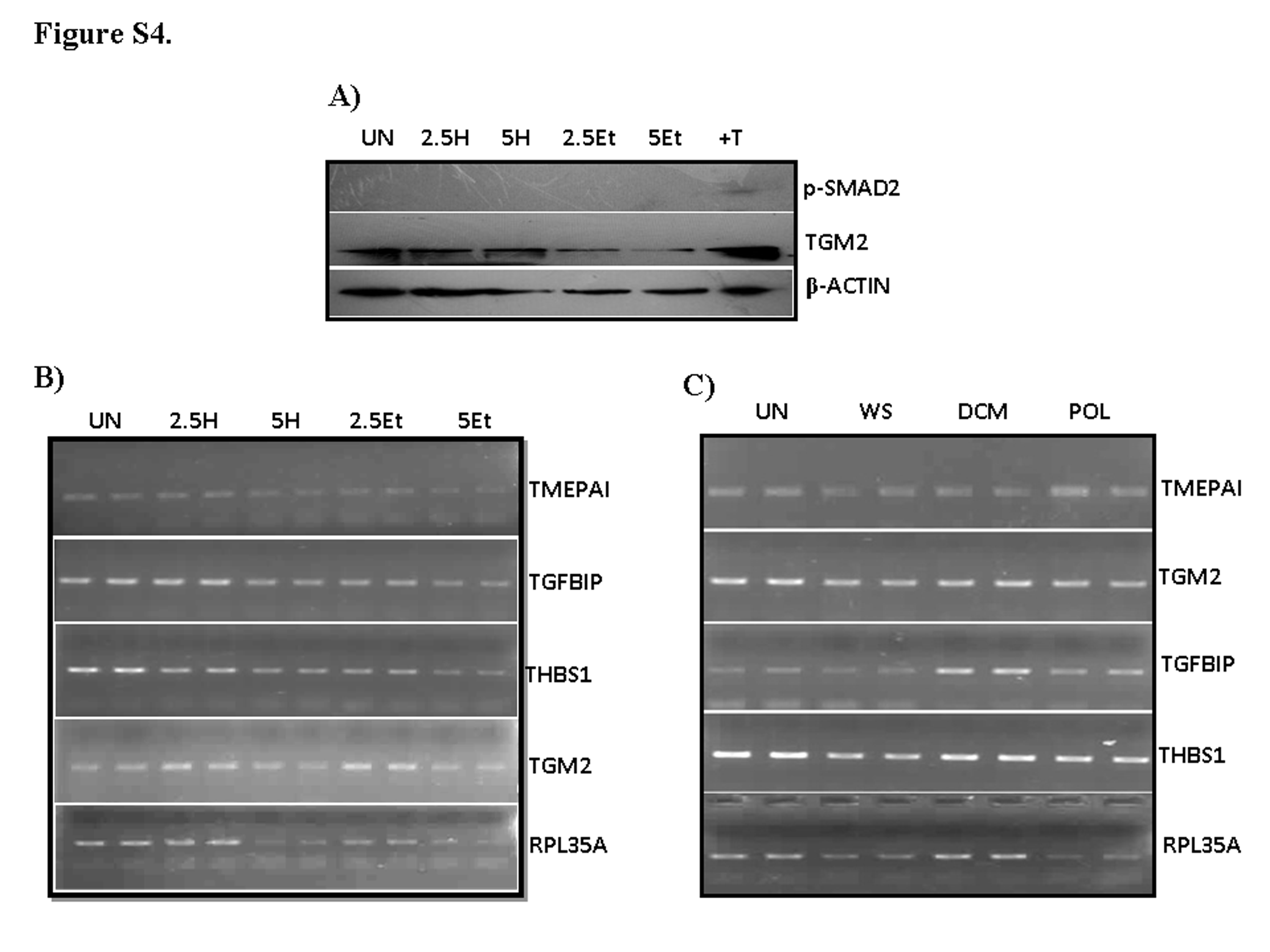

Supplement: Figure S4 — Arecanut induces TGF-β signaling in foreskin keratinocytes through TGF-β. Human Foreskin keratinocytes cells were serum starved for 24 hours and treated with either areca nut water (H) or ethanol (Et) extracts 2.5, 5 µg/ml (2.5H/5H & 2.5Et/5Et) in serum free medium for 48 hours. A-D) qRT-PCR analysis showing expression of TGF-β down-stream target genes TGM2, TMEPAI, THBS1, TGFBI and ALK5 (TβRI) inhibitor (SB 431542) reverses the Areca nut induced expression of the above genes. Treatment of human Foreskin keratinocytes cells with both the Alkaloid and Polyphenol fractions of areca nut water extract induced TGF-β down-stream target as shown by Real Time PCR (Figure S4E-H) and induction of genes by alkaloid and polyphenol fractions of areca nut was compromised in presence of TβRI inhibitor. UN-Untreated, 2.5H & 5H− 2.5 and 5 µg/ml Areca nut water extract, 2.5 Et,& 5Et- 2.5 and 5 µg/ml, ALK5- ALK5 inhibitor, T-TGF-β, WS- water supernatant, POL- Polyphenol supernatant, DCM- Dichloromethane fraction. (*** = P<0.0001, compared to untreated). (TIF) [file pone.0051806.s004.tif]

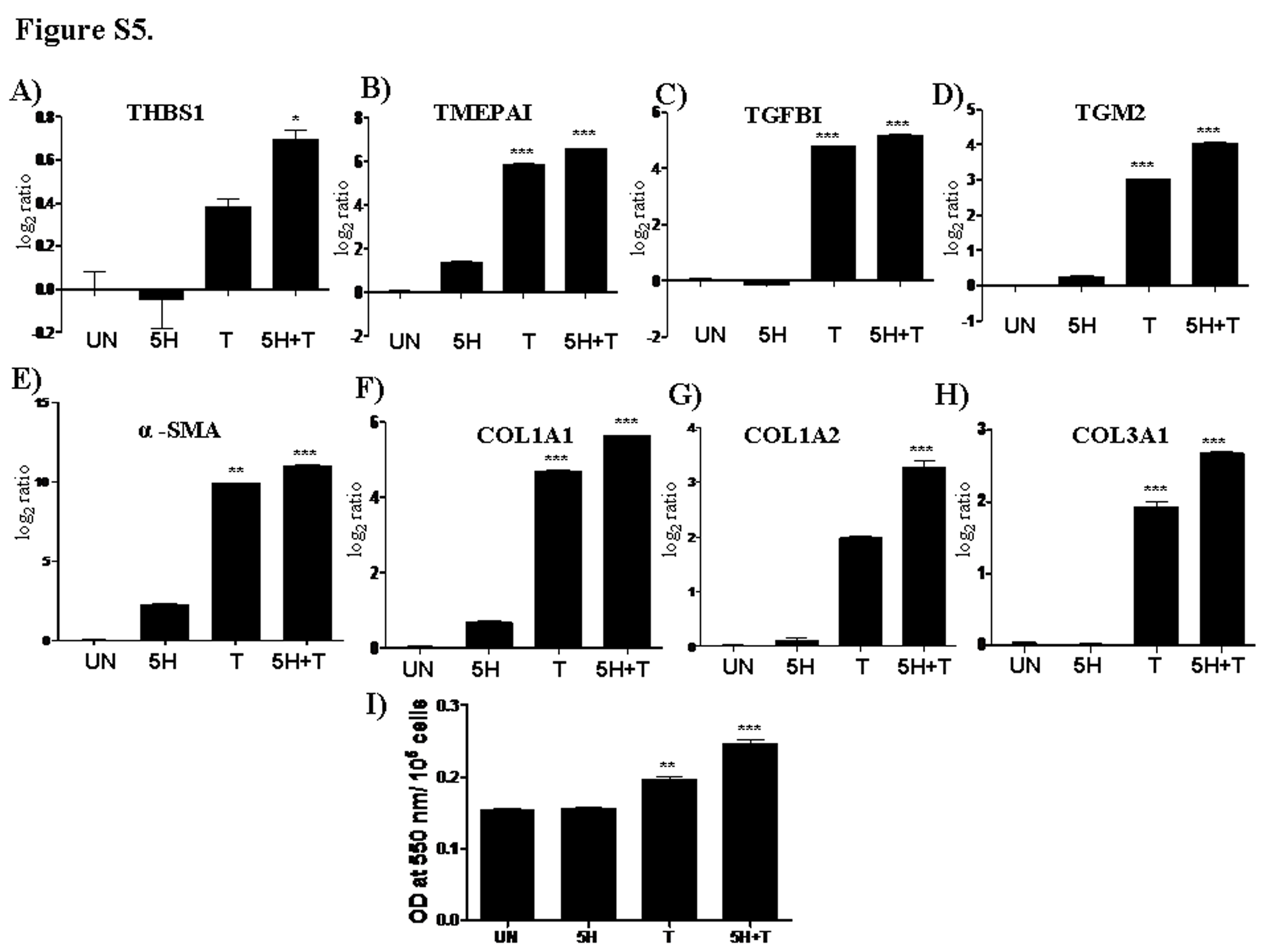

Supplement: Figure S5 — Areca nut potentiates TGF-β action on FF cells. Human foreskin fibroblast (FF) cells were treated for 72 hours with areca nut water extract (5H− 5 µg/ml) and or TGF-β (5 ng/ml) after 24 hrs of serum deprivation in 0.2% serum and the expression of genes were looked at by Real-Time PCR (Figure S5 A–H). FF cells were treated with areca nut water extract and or TGF-β for 3 days and stained with “Direct Red 80” for total collagen. Figure S5 I shows the quantitation of the Direct Red staining as measured at 550 nm. (TIF) [file pone.0051806.s005.tif]
